# Highly Sensitive Detection and Differentiation of Endotoxins Derived from Bacterial Pathogens by Surface-Enhanced Raman Scattering

**DOI:** 10.3390/bios11070234

**Published:** 2021-07-11

**Authors:** Xiaomeng Wu, Yiping Zhao, Susu M. Zughaier

**Affiliations:** 1 College of Food Science and Nutritional Engineering, National Engineering Research Center for Fruit & Vegetable Processing, Key Laboratory of Fruit & Vegetable Processing, Ministry of Agriculture, Beijing Key Laboratory for Food Non-Thermal Processing, China Agricultural University, Beijing 100083, China; wuxmeng@cau.edu.cn; 2 Department of Physics and Astronomy, University of Georgia, Athens, GA 30602, USA; 3 Department of Basic Medical Sciences, College of Medicine, QU Health, Qatar University, Doha P.O. Box 2731, Qatar; 4 Biomedical and Pharmaceutical Research Unit, QU Health, Qatar University, Doha P.O. Box 2731, Qatar

**Keywords:** SERS, LPS, bacteria, endotoxin, lipid A, silver nanorods

## Abstract

Bacterial endotoxins, as major components of Gram-negative bacterial outer membrane leaflets and a well-characterized TLR4-MD-2 ligand, are lipopolysaccharides (LPSs) that are constantly shed from bacteria during growth and infection. For the first time, we report that unique surface-enhanced Raman scattering (SERS) spectra of enteric LPSs from *E. coli*, *S. typhimurium*, *S. minnesota*, *V. cholerae*, *Rhizobium* species *R*. CE3, and *R*. NGR, as well as *Neisseria meningitidis* endotoxin structures, LPSs, lipid A, and KDO2-lipid A can be obtained. The characteristic peaks of the SERS spectra reveal that most of the tested LPS structures are from lipids and saccharides, i.e., the major components of LPSs, and these spectra can be successfully used to differentiate between endotoxins with principal components analysis. In addition, all the LPS samples here are measured at a concentration of 10 nmole/mL, which corresponds to their relevant pathophysiological concentrations in clinical infections. This study demonstrates that LPSs can be used as biomarkers for the highly sensitive detection of bacteria using SERS-based methods.

## 1. Introduction

**Figure A1:**
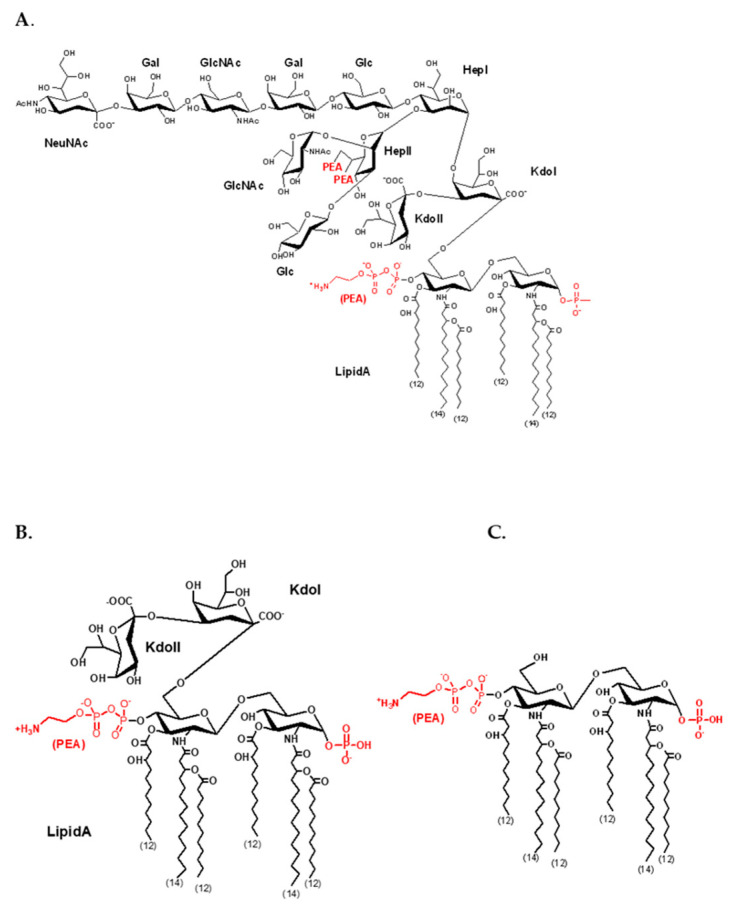
Schematic representation of meningococcal LPS structures. (**A**) *N. meningitidis* strain NMB wild-type LPS structure; (**B**) minimally glycosylated meningococcal KDO_2_-lipid A structure; (**C**) unglycosylated meningococcal lipid A structure.

An endotoxin or lipopolysaccharide is a glycolipid and is a major component of the outer membrane in Gram-negative bacteria. Endotoxins are shed from live bacteria as membrane blebs and vesicles or released from dead bacteria into tissue at the site of infection. LPSs are well-characterized pathogen-associated molecular pattern (PAMP) ligands that bind to the human TLR4-MD-2 receptor and elicit strong proinflammatory responses in immune cells [1,2]. For example, *Neisseria meningitidis* is a strictly human pathogen that causes meningitis and is the leading cause of fulminant sepsis and death [3]. The meningococcal LPS molecule produced by *Neisseria meningitidis* is composed of a lipid A component containing di-glucosamine that is linked to two KDO sugars and a heptose containing sugar or saccharide chain, as shown in Figure A1. The meningococcal LPS is a very potent inducer of proinflammatory mediator release, known as the cytokine storm, which contributes to massive fulminant meningococcal sepsis and rapid death [3]. As such, the rapid and sensitive detection of trace amounts of meningococcal LPSs in biological fluids is a highly desirable approach to help diagnose meningococcal infection, thus facilitating therapy and saving lives.

**Figure A2:**
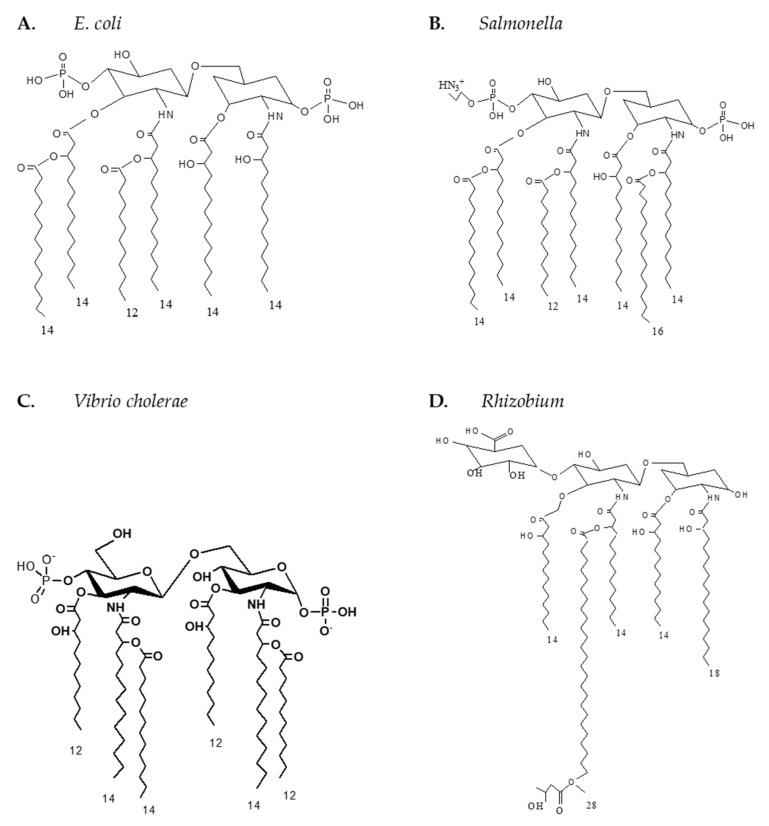
Schematic representation of different enteric lipid A structures. (**A**): *E. coli*; (**B**): *Salmonella*; (**C**): *Vibrio cholerae*; (**D**): *Rhizobium*.

Similarly, other Gram-negative bacteria contain endotoxins or LPSs as a major component of their outer membrane. Enteric bacteria LPS structures also contain a lipid A moiety linked to repeating units of polysaccharide chains, known as the O-antigen. Similar to meningococcal LPSs, enteric KDO_2_-lipid A is responsible for the endotoxic activity of LPSs; however, LPS and lipid A structures from enteric pathogens are more diverse and vary greatly in their ability to elicit TLR4-MD-2 mediated inflammatory responses [4]. Enteric lipid A structures, such as *E. coli*, *Salmonella*, and *Klebsiella*, all have fatty acyl chain lengths ranging from 10 to 16 carbon atoms, as shown in Figure A2, while other Gram-negative bacteria like *Bacteroides* and *Rhizobium* contain branched fatty acyl chains with extended lengths of up to 28 carbon atoms [5]. The length of the fatty acyl chain impacts lipid A endotoxic activity, where extended length fatty acyl chains dramatically reduce lipid A potency and act as a TLR4-MD-2 antagonist rather than as an agonist. These pathogenic Gram-negative bacteria can shed active endotoxins and elicit pro-inflammatory responses. Consequently, the rapid and sensitive detection of enteric endotoxin structures in biological fluids and consumed food would greatly restrict the transmission of infections.

The current methods to detect endotoxins in biological fluids and environmental samples are often time-consuming and costly, and some methods are not sensitive enough. A limulus amebocyte lysate (LAL) assay is the most common method to detect an endotoxin [6]. The LAL assay depends on the enzyme purified from a horseshoe crab that forms a clot upon detecting an endotoxin [6]. In spite of the significant enhancement of enzyme specificity and LAL assay performance, the method may yield false positive or negative results as the enzyme reacts with abundantly available glucan molecules derived from yeast and plant sources [6]. Matrix-assisted laser desorption ionization/time of flight mass spectroscopy (MALDI-TOF MS) has also been used to identify bacterial endotoxins accurately, but this method is very expensive and time-consuming and requires specific expertise [7]. New methods that can rapidly and reliably detect bacterial endotoxins with a low limit of detection are highly desired in the fields of human health, environmental monitoring, and food safety.

In recent years, with the development of nanotechnology, biosensors based on novel nanostructures have been used to detect and identify trace amounts of endotoxins in human fluid samples using fluorescence, chemiluminescence, and electrical gradient applications [8,9,10]. Surface-enhanced Raman scattering (SERS) is considered to be one of the most sensitive analytical tools, with the potential to perform single-molecule detection under ambient conditions [11]. Recent ultra-sensitive SERS-based methods can detect single molecule reactions on a substrate surface [12,13]; however, Raman-based studies on the unique spectra of bacterial endotoxin are currently very limited. A recent study reported the in situ detection of a *Pseudomonas aeruginosa* endotoxin using a nanogold-sputtered cicada wing as a SERS chip; however, it used 4-mercaptobenzoic acid (4-MBA) and p-aminophenol (PAP) as SERS reporters rather than the intrinsic SERS spectra of the endotoxin itself [14]. In order to establish a highly sensitive SERS platform for intrinsic endotoxin detection, highly reproducible and practical substrates are essential. Our previous studies have demonstrated that silver nanorods (AgNRs) fabricated by oblique angle deposition can act as a highly sensitive and reproducible substrate with a SERS enhancement factor of ~10^9^ and a batch-to-batch variation of <10% [15]. AgNR substrates have been applied in the detection of viruses, pathogenic bacteria such as *E. coli* O157:H7, *Salmonella typhimirium*, and *Staphylococcus aureus*, and toxins such as aflatoxins [16]. The utility of SERS from silver nanorods (AgNRs) for the rapid detection of bacterial pathogens via biomarkers has been demonstrated. We have reported that SERS can rapidly detect *Pseudomonas aeruginosa* pigment, pyocyanin, and pyoverdine in biological fluids with very high sensitivity and specificity when using a AgNR substrate [17]. 

Here, we report a proof-of-concept study in which SERS from AgNR is utilized to investigate the endotoxin structures of pathogenic bacteria. Highly purified and defined endotoxins from *N. meningitidis* and other pathogenic enteric bacteria are used to determine the unique SERS spectra. Utilizing principal component analysis (PCA), the unique SERS fingerprint spectra are able to accurately distinguish between meningococcal LPS structures and a collection of enteric LPS structures. 

## 2. Materials and Methods

For endotoxin (LPS) sample preparation, the *Neisseria meningitidis* lipooligosaccharide (herein called LPS) and its truncated LPS structures (KDO_2_-lipid A and unglycosylated lipid A) were isolated from *N. meningitidis* serogroup B and its isogenic mutants as described previously [2]. Of note, the *Neisseria meningitidis* endotoxin structure contains one side saccharide chain, hence referred to as lipooligosaccharide (LOS), which was used to distinguish between other lipopolysaccharide (LPS) structures. Purified LPSs from the enteric bacteria *E. coli*, *Vibrio cholerae*, *Salmonella typhimurium*, and *Salmonella minnesota* were obtained from Sigma (St. Louis, MO, USA) and further purified and quantified based on the lipid A content as previously described [4]. Briefly, residual membrane phospholipids were removed by the repeated extraction of the dried LPS samples with ethanol and water in a ratio of 9:1. The expected fatty acyl components of 3-OHC12:0, 3-OHC14:0, and C12:0 and the absence of membrane phospholipids were assessed by mass spectroscopy (GC-MS) (Dr. Russell Carlson, Complex Carbohydrate Research Center, University of Georgia, Athens, GA). LPS stock solutions were prepared in pyrogen-free water at a 10 nmole/mL concentration with extensive vortexing and sonication prior to each dilution as described previously [4]. Highly purified LPSs from *Rhizobium etli* strain CE3 and *Rhizobium niger* strain NGR (obtained as kind gifts from Dr. Russell Carlson, Complex Carbohydrate Research Center, University of Georgia, Athens, GA) were used at 10 nmole/mL concentrations. Lipid A is the major component of endotoxin structure responsible for biological activity. As such, all endotoxin structures used in this study were quantified based on their lipid A fatty acyl chain content rather than the total weight of molecule with and without saccharides chains, and stocks are made at 10 nmole/mL as described above [4]. 

Regarding silver nanorod (AgNRs) substrate fabrication, the AgNRs substrates used in this study were fabricated by an oblique angle deposition (OAD) technique that has been described previously [15,16,18]. Briefly, microscopic glass slides (BD, Portsmouth, NH) were cleaned with a piranha solution (80% sulfuric acid, 20% hydrogen peroxide, *v*/*v*) and rinsed with deionized (DI) water before being air-dried and loaded into a custom-made electron beam evaporation system. The glass slides were deposited with a 20-nm titanium layer and then a 200-nm silver film layer through evaporation at rates of ~0.2 nm/s and 0.3 nm/s, respectively. The slides were monitored in situ by a quartz crystal microbalance (QCM). To fabricate the AgNRs, the substrates were tilted to 86° with respect to the incident vapor, and silver was then deposited rate of ~0.3 nm/s until a thickness of 2000 nm was obtained. The morphology of the AgNR substrate has been reported previously [19,20,21]. According to a previous study, the AgNR substrate has a broad and strong absorption when the wavelength of light is >500 nm [19,20,21]. The uniformity and reproducibility of the AgNR substrate for SERS measurements have been reported to be smaller than 10% for the spot-to-spot variation and less than 15% for batch-to-batch variation [19].

Regarding SERS measurement and data analysis, 2 µL of the testing sample was dispensed directly on the AgNR substrate and vacuum-dried in the custom-made vacuum chamber to shorten the sample preparation time. The dried samples on the substrates were then measured by an Enwave ProRaman-L-785A2 Raman analyzer (Enwave Optronics, Irvine, CA, USA) with a 785 nm near-IR diode laser as the excitation source. SERS spectra were collected in a wavenumber range from 400 cm^−1^ to 1800 cm^−1^ for 10 to 30 s with a laser power on the sample of 100 mW. Spectra data were acquired from nine randomly selected spots on the AgNR substrate and showed good reproducibility (Appendix C). The original SERS spectra contain a broad fluorescence background, and thus all spectra baselines were first corrected and the average spectra from the nine different measurements per sample were presented as the final spectra results.

Data analysis was performed using version 9.0 of the Origin software package (OriginLab Corporation, Northampton, MA, USA). PCA was conducted with MATLAB 2000b (The MathWorks, Inc., Natick, MA, USA) using the PLS toolbox (Eigenvector Research, Inc., Wenatchee, WA, USA). Savitzky–Golay derivation, normalization, and the mean-center process were used to treat the raw spectra data prior to PCA analysis.

## 3. Results and Discussion

### 3.1. Meningococcal SERS Spectra

**Figure 1:**
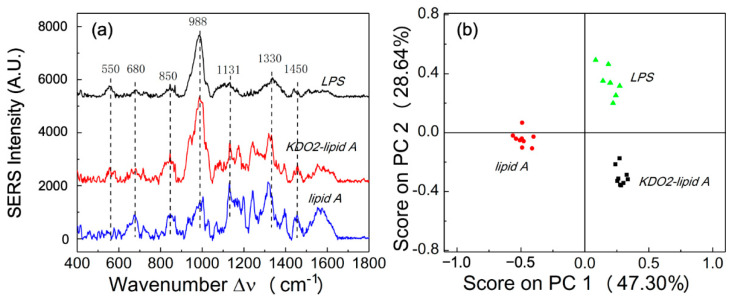
Meningococcal LPS Raman SERS spectra. (**a**) SERS spectra of LPS, KDO_2_-lipid A, and lipid A obtained from *Neisseria meningitidis* endotoxin structures. (**b**) The PCA score plot of SERS spectra of LPS, KDO_2_-lipid A, and lipid A.

In order to understand the effect of a LPS contributing to its unique SERS spectra, a *Neisseria meningitidis* LPS (wild type, denoted as LPS), *Neisseria meningitidis* truncated LPS (KDO_2_-lipid A), and *Neisseria meningitidis* unglycosylated lipid A (lipid A) at concentration of 10 nmole/mL were used to obtain SERS spectra. As shown in Figure 1a, the SERS spectrum of a LPS shows significant Raman shift peaks at Δ*ν* = 989 cm^−1^ and Δ*ν* = 1330 cm^−1^, whereas the SERS spectra of KDO_2_-lipid A has the same significant peaks as those observed from wild-type LPS spectra. Lipid A, the fatty acyl chain linked to the disaccharide backbone of LPS, is primarily responsible for the endotoxic activity in the human host, rather than the sugar chains in LPS [2]. When comparing the SERS signals from naked lipid A structure to those of the LPS with saccharide chains attached, the resulting SERS spectra suggest that the lipid A portion of the LPS could contribute significantly to its unique SERS signal. The SERS spectrum of KDO_2_-lipid A shows a relatively strong peak at Δ*ν* = 989 cm^−1^ and a weak peak at Δ*ν* = 1330 cm^−1^ as compared to those for lipid A, which indicates that the characteristic peak at Δ*ν* = 989 cm^−1^*,* which may come from carbohydrates [22]. The peak at Δ*ν* = 1330 cm^−1^ is typically assigned to δ(CH) in phospholipids, which is often used in the in vivo study of biological tissues [23]. The SERS spectra of lipid A have a relatively lower peak at Δ*ν* = 989 cm^−1^ and a much higher peak intensity at Δ*ν* = 1330 cm^−1^, which further suggests that the peak at Δ*ν* = 989 cm^−1^ is corresponding to the carbohydrates since KDO molecule is a sugar, and the signal from phospholipids is dominant in SERS spectra (Figure 1a). Similarly, the peak at Δ*ν* = 1131 cm^−1^, which was assigned to the mode of fatty acid, which also dominates the SERS spectra of lipid A [22,24]. Detailed SERS peak assignments are listed in Table 1. 

The data suggest that the SERS spectra from different meningococcal LPS structures can be distinguished based on their detailed compositions. To better analyze the data, we have also performed the PCA analysis as shown in Figure 1b. The spectra of *Neisseria meningitidis* LPS, KDO_2_-lipid A, and naked lipid A are well separated based on the score plots of PC1 (47.30%) and PC2 (28.64%). This study demonstrates the detection of highly purified meningococcal endotoxin structures and the differentiation of their SERS spectra in relation to the biological activity in a human host [2,4]. As previously reported, full-length LPSs and KDO_2_-lipid A show potent immune stimulatory activity in human macrophages, whereas naked lipid A is a very weak endotoxin [2]. The biological activities of endotoxin structures are correlated with fulminant sepsis and disease outcomes [25,26]. Therefore, SERS can be used for the rapid detection and differentiation of a meningococcal endotoxin, which may serve as an indicator of *Neisseria meningitidis* infection.

**Table 1:** SERS peak assignment for *Neisseria meningitidis* LPS structures.

Observed SERS Shift Δ*ν* (cm^−1^)	Vibrational Mode Assignment [22,27,28]
550	β(CH_2_) in ring
680	δ(C-O-C); fatty acid;
850	ν(C-O-C); saccharides (1,4 glycosidic link)
988	β(CH); carbohydrates
1131	ν(C-C); fatty acid
1330	δ(CH); phospholipid
1450	α(CH_3_/CH_2_), β(CH_3_/CH_2_)

β, bending; ν, stretching; δ, deformation.

### 3.2. Enteric LPS SERS Spectra

**Figure 2:**
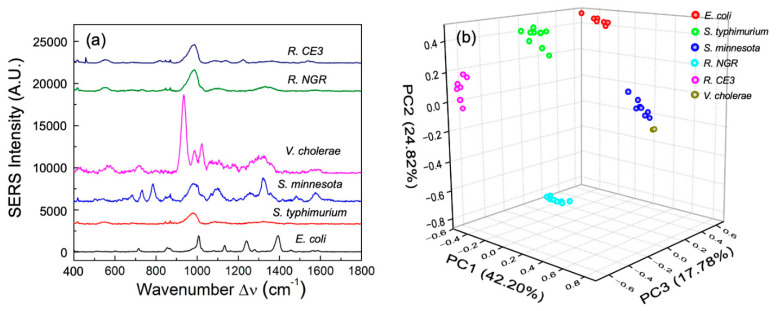
Different enteric LPS Raman SERS spectra. (**a**) SERS spectra of different enteric LPSs obtained from *E. coli*, *S. typhimurium*, *S. minnesota*, *V. cholerae*, *R*. CE3, and *R*. NGR. (**b**) PCA score plot of six enteric LPSs based on their SERS spectra.

The SERS spectra of enteric LPSs at equal molar concentrations of 10 nmole/mL from *E. coli*, *S*. *typhimurium*, *S. minnesota*, *V. cholerae*, *R*. NGR, and *R*. CE3 are shown in Figure 2a. The spectra of *S. typhimurium* and *S. minnesota*, which are from the same species (*Salmonella*), show similar peaks at Δ*ν* = 543 and 981 cm^−1^, which can be signed to the saccharide and lipid [22,24,27,29]. Similarly, the SERS spectra of *R*. CE3 and *R*. NGR also show the same peaks at Δ*ν* = 552, 981, and 1330 cm^−1^ (Figure 2a), which represents cholesterol and lipids [22,24,27,28,29,30]. The spectra of *E. coli* and *V. cholerae* are entirely different from the spectra of the *Salmonella* LPS and *Rhizobium* LPS, which indicates that unique the SERS spectra of LPS structures could be used to differentiate bacteria species (Figure 2a). Detailed SERS peak assignment for enteric and *Rhizobium* LPS are shown in Table 2. Note that most of the spectral peaks of these LPSs are from lipids and carbohydrates, which are the major components of LPS. The 3D-score plot of PC 1 (42.20%), PC 2 (24.82%), and PC 3 (17.76%) for the enteric and *Rhizobium* LPS based on the SERS spectra are shown in Figure 2b. All the spectra from the same LPS are grouped together well and are evidently separated from one another. Different bacteria express unique LPS structures that do not change when expressed in viable cells or shed from the membrane. Therefore, unique LPS SERS can be used to distinguish various bacteria strains.

The SERS spectral peak locations are usually referred to as fingerprint spectra. Different molecules have different fingerprint spectra, which can be used to differentiate between different molecular structures [16]. It is known that LPSs from different bacteria have different molecular structures and bonds [4]. Therefore, our data analysis focused on the peak location difference rather than intensity or peak shape. Since all spectra were normalized, the peak intensities were less important when comparing various LPS structures. In our study, the PCA method was employed to depict the differences between peak intensity, peak position, relative intensity and spectral shape (Figure 2B). Although minor structural differences between bacterial LPSs were found, their biological activities were different and correlated to their effect on human host response [4]. For example, *Rhizobium* species that contain extended length fatty acyl chains do not exert immune stimulatory activity on human macrophages, which is in contrast to other LPSs like *E. coli*, *Salmonella* and *Vibrio* with fatty acyl chain lengths of C12 and C14. Moreover, *Salmonella* and *E. coli* LPS structures vary in their biological activity and differential induction of human TLR4 signaling pathways, consequently modulating the outcome of host response [4]. Taken together, the data provide a proof-of-concept for the utility of SERS in rapid detection of endotoxin and in distinguishing endotoxin structures from bacterial pathogens. 

## 4. Conclusions

In this work, we have shown that the LPS, KDO_2_-lipid A, and lipid A of *Neisseria meningitidis* endotoxin structures, as well as the enteric LPSs from *E. coli*, *S. typhimurium*, *S. minnesota*, *V. cholerae*, *R.* CE3, and *R.* NGR, exhibit distinguishable fingerprint SERS spectra that can be separated well using a conventional PCA method. The difference in SERS spectra originates from the carbohydrates and phospholipids of different LPS structures. All the SERS measurements were carried at a low concentration (10 nmole/mL) in accordance with the relevant pathophysiological concentration in clinical infection, which means that the SERS technique has a great potential to detect and differentiate physiologically relevant enteric LPSs from biological fluids. In addition, the spectral differences of the different LPSs demonstrate that LPSs can be used as biomarkers for detecting bacteria, especially in terms of SERS-based detection, which could potentially lead to new ways to sense bacteria.

## Figures and Tables

**Table 2:** SERS peak assignment for enteric LPS structures.

Observed SERS Shift Δ*ν* (cm^−1^)	Vibrational Mode Assignment [22,27,30,31,32]
543	δ(C-O-C) in glycosidic ring
552	β(CH_2_) in ring
715	ν(C-N)
733	β(C-O-C) in carbohydrates
787	ν(C-O) in ring
855	δ(C-O-C)
981	β(CH) in lipid
1025	ν(CO) in carbohydrates
1087	ν(C-O) in lipid
1131	ν(C-C) in fatty acid
1309	τ(CH_3_/CH_2_) in lipid
1330	δ(CH) in phospholipid

β, bending; δ, deformation; τ, twisting; ν, stretching.

**Figure A3:**
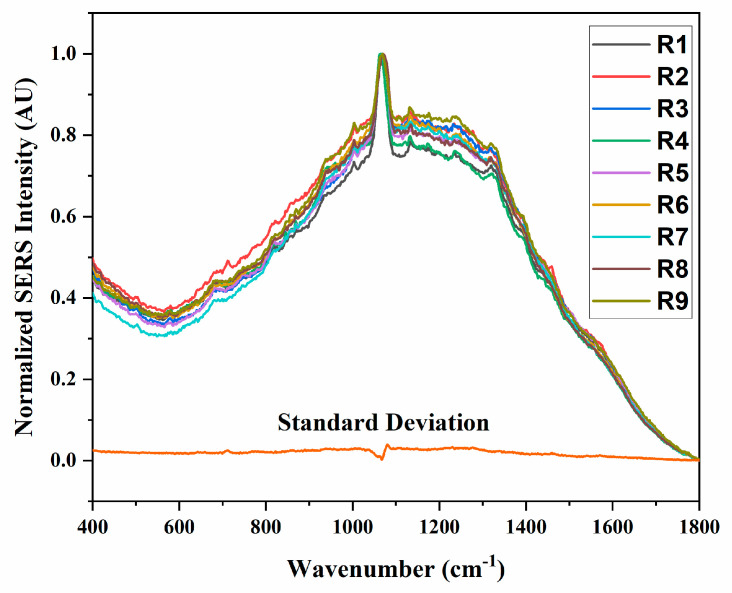
Reproducibility of LPS spectra on AgNR. Nine spectra (R1 to R9) are shown for the same LPS as measured at different locations on the AgNR substrate.

## Data Availability

Available upon request.
